# Associations among mental well-being, marital quality, and maternal depressive symptoms in China: a cross - sectional study and mediated analysis

**DOI:** 10.3389/fpsyt.2025.1551588

**Published:** 2025-05-23

**Authors:** Wang Jinhuan, Yu Jie, Sun Ying, Wei Dongzhuo, Geng Zhongli, Wang Jiayue, Wang Yan, Ren Jintao

**Affiliations:** ^1^ Department of Tieling Mental health Prevention and Control, The Third People’s Hospital of Liaoning, Tieling, China; ^2^ Department of Ophthalmology, The Fourth People’s Hospital of Shenyang, Shenyang, China; ^3^ Beijing Anding Hospital, Capital Medical University, Beijing, China; ^4^ Department of Shenyang mental Health Prevention and Control, Shenyang Mental Health Center, Shenyang, China; ^5^ Department of First Clinical, Jinzhou Medical University, Jinzhou, China; ^6^ Center for Psychological Development, China Medical University, Shenyang, China

**Keywords:** postpartum depression, mental well-being, marital quality, mental health, China

## Abstract

**Background:**

Postpartum depression (PPD) is an extremely common mood disorder that occurs at any time up to 1 year after delivery. PPD can have a negative impact on the mother and family. This study aimed to determine the prevalence of PPD and factors influencing PPD. Specifically, this study focused on the effects of marital quality and mental well-being on PPD and the mediating roles

**Methods:**

A cross-sectional study was conducted in the Maternity and Child Health Care Hospitals of Liaoning Province in northeast China. The PHQ-9 scale was used to screen for PPD with a score ≥10, indicating a positive result. The Warwick-Edinburgh Mental Health and Marriage Perception scales were used to evaluate the mental well-being and marital quality of parturients.

**Results:**

A total of 1048 participants were included in the study. The prevalence of PPD symptoms was 11.16%. Factors, such as education level (*F* = 2.63; *p* < 0.05), co-living status (*F* = 5.84; *p* < 0.01), agreement with fetal gender expectations (*t* = 19.39; *p* < 0.001), amount of physical activity (*F* = 17.15; *p* < 0.001), and knowledge about PPD (*t* = 3.66; *p* < 0.001 and *t* = 5.099; *p* < 0.001), were all associated with the PPD score and the prevalence. Mental well-being and marital quality were intricately linked to PPD symptoms. Mental well-being influenced PPD via two mediating factors (marital interaction [*p* < 0.001] and marital conflict [*p*< 0.001]).

**Conclusions:**

PPD is a significant postpartum issue that is influenced by numerous factors. Early screening of parturients, accurate diagnosis of PPD, and timely intervention are crucial. Targeted interventions addressing risk factors may help to mitigate the incidence of PPD.

## 1 Introduction

Changes in the physiologic-psychological-social environment are known to occur during the perinatal period (1). Such changes have an impact on the mental well-being of gravidas and parturients, thereby elevating the susceptibility to various mental disorders (2), most notably anxiety and depression (3). Postpartum depression (PPD) is an extremely common mood disorder that occurs at any time up to 1 year after delivery and can have a negative impact on the mother and the family (4). Approximately 1 in 7 women (13%) are affected by PPD. Research suggests that PPD should receive given special attention in the puerperium (5). A 2022 cross-sectional study in Palestine reported a postpartum depression (PPD) prevalence of 33.9% at 7–12 weeks postpartum, with factors such as husband’s education level, primiparity, vacuum use, stressful events during pregnancy, and low social support significantly associated with PPD (6). Another study found PPD prevalence rates of 22.4% at 3 days and 3.2% at 6 months postpartum (7). A multinational study revealed an overall PPD symptom rate of 13.6%, ranging from 2.3% in Syria to 26% in Ghana, with an overall diagnosis rate of 6.2% (8).

Studies have shown that perinatal depression can last a long time, and if not effectively treated, can have a negative impact on maternal health, the family, and the baby (9). Interestingly, most women with perinatal depression refrain from seeking professional assistance (10).

The clinical practice guidelines of the American College of Obstetricians and Gynecologists (ACOG) and Chinese Expert Consensus suggest the need to screen women for mental health issues, including anxiety, depression, stress, suicide, and self-injury, during the perinatal period (11, 12). The recommended screening tools for depressive symptoms include the Patient Health Questionnaire-9 (PHQ-9) and Edinburgh Postpartum Depression Scale (EPDS) (13). Several confirmatory studies have utilized these tools extensively. The purpose of screening is to fulfill the requirements of tertiary prevention by detecting and intervening at the earliest possible time to minimize pain and burden (14). Randomized controlled trials have shown that APP-based cognitive behavioral therapy programs are effective in preventing PPD during the early postpartum period (15).

Several biological, psychological, socioeconomic, and cultural factors have associations with the development of PPD (16), including depression, anxiety insomnia, and stress (16, 17), infant feeding (18), unplanned pregnancy, a first-time mother, a poor mother-in-law relationship, and poor family support (19).

Negative partner or family relationship (20), poor self-esteem, and a miscarriage (21). Mobile phone usage, low birthweight (21), employee status (22), financial conditions (23), negative life events, and neuroticism (24). Robust social support and regular physical exercise serve as protective factors against PPD, a conclusion supported by substantial empirical evidence (25, 26). There are distinctive risk factors that impact PPD symptoms in China. For example, girl infants exhibit a higher prevalence of depressive symptoms compared to boys, which could potentially be attributed to the cultural inclination towards male offspring in China (27, 28). The practice of “zuoyuezi,” also known as “doing the month,” is a distinctive form of postnatal self-care in China. Zuoyuezi has been shown to provide social support and reduce depressive symptoms for mothers receiving care (29), while simultaneously leading to increased conflicts when receiving care from a mother-in-law, resulting in higher levels of depressive symptoms (30, 31).

PPD has been shown to negatively influence the physical and mental well-being of women (32). Partner support and the quality of a couple’s relationship are correlated with PPD (33) and intimate partner violence (34). However, there have been no studies involving the relationship between marital happiness and PPD symptoms. We propose that mental well-being and marital quality influence PPD symptoms through a mediating relationship.

With the social economy advances in China, mental health issues have gained significant attention. Comprehensive planning has been made for the entire population, ranging from severe mental disorders to the establishment of a social psychological service system (35). According to several official government documents, mental health screening for perinatal women is considered one of the key tasks in promoting mental health among specific populations. A specific plan for screening and intervention of women during the perinatal period was formulated in the annual major public health service projects in China. Maternal healthcare institutions and primary healthcare institutions are required to include screening for maternal depression during routine prenatal examinations and postpartum visits. Trained medical personnel or social workers will perform screening and pregnancy and PPD follow-up evaluations. Our research, as one part of the region, received support from this plan.

We performed a cross-sectional survey of parturients to document the prevalence of PPD symptoms. We then examined the associations between marital well-being, positive psychology, and PPD symptoms. Next, we analyzed the mediating role of positive psychology on the effect of marital well-being on PPD.

## 2 Materials and methods

### 2.1 Participants

The cross-sectional study was administered from 13 May to 31 December 2023. Convenience sampling was adopted to recruit parturients in four Maternity and Child Health Care Hospitals of Liaoning Province. Inclusion criteria: participants must be 18 years of age or older; within a few days to 12 months postpartum; voluntarily signed the informed consent form. The exclusion criteria were as follows: <18 years of age; declined to participate; multiple pregnancies; a current or prior history of bipolar disorder, schizophrenia, or other mental illnesses; known mental disorders; a major chronic illness; cognitive impairments;visual or auditory processing disorders;incomplete questionnaires; questionnaires with clear logical inconsistencies and obstetric complications (including but not limited to severe preeclampsia/eclampsia, placenta previa, placental abruption, major postpartum infection, intrauterine fetal demise, major congenital anomalies, or newborn weight <1500 grams).

### 2.2 Procedures

The approval for this study was obtained from the Medical Ethics Committee of Liaoning Mental Health Center and Hospital in Liaoning, China (Approval number: LNMHC2023-TT0001). A cross-sectional survey was performed and online questionnaires were made using an online survey platform (www.wjx.cn). Questionnaires were administered via QR codes placed in the outpatient area of the maternity hospital. Participants completed the questionnaires during waiting periods for follow-up visits or vaccinations, under the supervision of medical staff. The participants completed the questionnaires with unified instruction and affirmed comprehension of the study purpose. Informed consent was obtained from the participants and the validity of the collected questionnaire was checked. The responses were considered invalid for the following reasons: the response time was either too short or too long; the response was repeated or abnormal; the IP address was a duplicate or abnormal; the response consistency test failed; and if the questionnaire did not pass the “false question” test. A total of 1148 valid questionnaires were collected and the effective recovery rate was 84.3%.

### 2.3 Measurement tools

#### 2.3.1 Associated characteristics survey

Data on associated variables,including 1)demographic:years of marriage, educational level, only child family, monthly income;location, cohabitation, working consistently during pregnancy, postpartum childcare centers? 2)health-related characteristics:smoking,alcohol consumption;exercise; 3)obstetric, current pregnancy, and infant-related characteristics, and their association:planned pregnancy;mode of conception;number of pregnancies;number of deliveries; adverse pregnancy history;high-risk maternity;how many kids is that?fatal sex;postpartum stage;4)psycho and social characteristics:maternal fetal sex expectation,consistency in fetal sex expectation, key family members’ fetal sex expectation, literacy (Know postpartum depression? Know where seeking-help for postpartum depression).

#### 2.3.2 Patient health questionnaire-9 (PHQ-9)

The PHQ-9 is a self-report version based on 9 items from the DSM-IV diagnostic criteria of major depression disorder (36). The PHQ-9 has good reliability and validity in facilitating the diagnosis of depression in the general population (37)and parturients (38, 39). Ren et al. conducted a study of reliability and validity assessment in Chinese samples (37). The ACOG clinical practice guidelines recommend the PHQ-9 as one of the tools to screen parturients for depressive symptoms 8. Each item has a 4-point frequency score ranging from “0” (not at all) to “3” (almost every day). The total score ranges from 0–27. The higher the score, the more severe the depressive symptoms. The scoring ranges for different levels of depression severity are as follows: minimal or no depression (0–4);mild depression (5–9);moderate depression (10-14);moderately severe or severe depression (15-19) Cronbach’s alpha for the internal consistency reliability of the Chinese version of the PHQ-9 was 0.86 for the entire scale (40). The Cronbach’s α coefficient of the PHQ-9 was 0.892 for patients with MDD in psychiatric hospital (41). A score of 10 was the cut-off value for “positive depressive symptoms.”

#### 2.3.3 Warwick-Edinburgh mental health scale

The WEM-WBS was developed to enable monitoring of mental well-being. The WEM-WBS consists of 14 items covering hedonic and eudaimonic aspects of mental health, including positive affect, satisfying interpersonal relationships, and positive functioning. Individuals completing the scale are required to respond to statements that best describes their experience over the past 2 weeks.The Likert scale represents a score for each item ranging from 1 (never) to 5 (all of the time) for a minimum score of 14 and a maximum score of 70. The overall score for the WEM-WBS was calculated by determining the scores for each item with equal weights. A higher WEM-WBS score indicated a higher level of mental well-being (42). The Chinese version of the Warwick–Edinburgh Mental Well-being Scale was used as the reliability and validity basis for the Chinese study, Cronbach’s α = 0.94 and test–retest reliability = 0.83 (43).

#### 2.3.4 Marriage perception scale

Wang et al. developed the MPS, which is based on subjective perceptions of marriage. The MPS is used to assess the quality of an individual’s subjective feelings about marriage as indicators, which cover 3 dimensions, such as “Couple Interaction,” “Family Relationships,” and “Couple Conflict,” with 10, 5, and 5 questions for each factor, respectively. A Likert scale ranging from 1 (completely inconsistent) to 7 (completely consistent) was used. The factor score was calculated by summing up the corresponding item scores as follows: MPS total score = couple interaction + family relationship - couple conflict. A higher MPS total score indicated better marital quality with Cronbach’s α of 0.89 (44).

### 2.4 Statistics

The data were analyzed using online Social Science Statistics SPSSpro. Descriptive statistics, including frequency, percentage, and mean ± standard deviation, were employed to characterize the data. For comparisons between two groups, independent samples t-tests were utilized for normally distributed data, while the Mann-Whitney U test was applied for non-normally distributed data. In cases involving three or more groups, one-way ANOVA (assuming homogeneity of variance) was conducted for normally distributed data, whereas the Kruskal-Wallis test was used for non-parametric data. Chi-square tests were performed to examine differences in negative and positive symptoms of depression across demographic and psychosocial factors. Pearson correlation analysis was conducted to assess the relationships between PHQ-9 scores and both WEMWBS and MPS scores. Following a homogeneity of variance test, Welch’s ANOVA was selected to evaluate differences in WEMWBS and MPS scores across varying levels of PHQ-9 severity. The statistical analysis to examine the potential mediating effects of WEM-WBS and MPS on postpartum women’s PHQ-9 scores was conducted using a mediation analysis framework.To test this hypothesis, we conducted mediation analyses using WEM-WBS and MPS (including its sub-scales) as independent variables and mediators, respectively. We employed stepwise regression to examine the total effect and potential mediating pathways. Indirect effects were calculated, and their significance was assessed using the Bootstrap method. Based on the presence or absence of significant mediating effects, we constructed a structural equation model (SEM) to delineate the relationships among WEM-WBS, MPS (including sub-scales), and PHQ-9 scores, and tested the overall model fit and significance.The significance level was set at *p*<0.05.

## 3 Results

A total of 1148 pregnant women in northeast China were invited to participate in this study and complete the assessment. Overall, 1048 participants completed all assessments and provided effective answers. Participants who did not complete all assessments were regarded as non-responders (n = 100). The associated characteristics of this sample are shown in **Table 1**. The mean age of the participants was 30.171 years (SD ± 4.957 years). Among the 1048 participants, the prevalence of depression symptoms (PHQ-9 ≥ 10) was 11.15% (n = 117).

**Table 1:** Sociodemographic characteristics of women with PPD symptoms.

Variables	PHQ-9 (<10)	PHQ (≥10)	χ^2^	p
	931 (88.83)	117 (11.16)		
Demographic				
Years of marriage				
<1 year	148 (87.06)	22 (12.94)	1.07	0.784
1~2 years	326 (89.81)	37 (10.19)		
3~4 years	176 (88.00)	24 (12.00)		
≥5 years	281 (89.21)	34 (10.79)		
Educational level				
Primary and below	34 (73.91)	12 (26.09)	20.27	<0.001
Lower or upper secondary school	382 (86.62)	59 (13.38)		
Technical college	282 (93.07)	21 (6.93)		
Bachelor	216 (89.63)	25 (10.37)		
Postgraduate or above	17 (100)	0 (0.00)		
Only child family				
Yes	421 (90.93)	42 (9.07)	3.66	0.056
No	510 (87.18)	75 (12.82)		
Monthly household income				
≤3000 RMB	230 (88.12)	31 (11.88)	0.23	0.972
3001-4999 RMB	296 (88.89)	37 (11.11)		
5000-9999 RMB	326 (90.07)	40 (10.93)		
≥ 10000 RMB	79 (89.77)	9 (10.23)		
Location				
Rural	261 (86.14)	42 (13.86)	3.13	0.077
Urban	670 (89.93)	75 (10.07)		
Cohabitation				
Partner Only	624 (90.57)	65 (9.43)	10.6	0.014
Parents (or in-law)	233 (86.94)	35 (13.06)		
Long-distance husband and wife	40 (76.92)	12 (23.08)		
Others	34 (87.18)	5 (12.82)		
Working consistently during pregnancy				
Yes	205 (91.93)	18 (8.07)	2.73	0.098
None	726 (88.00)	99 (12.00)		
Health-related				
Smoking				
Never	862 (90.14)	105 (10.86)	2.22	0.329
Used to smoke, quitting	25 (80.65)	6 (19.35)		
Occasional smokers (more than once a month)	44 (88.00)	6 (12.00)		
Alcohol consumption				
Never	870 (89.60)	101 (10.40)	8.21	0.017
Used to drink, Cut out now	50 (78.13)	14 (21.87)		
Occasional drinkers (multiple times per month)	11 (84.62)	2 (15.38)		
Exercise				
Never	301 (85.27)	52 (14.73)	8.28	0.016
Occasionally (more than once a month)	446 (89.74)	51 (10.26)		
Regular (more than once a week)	184 (92.93)	14 (7.07)		
Obstetric, current pregnancy, and infant-related characteristics, and their association				
Planned pregnancy				
Yes	661 (90.92)	66 (9.08)	10.41	0.001
No	270 (84.11)	51 (15.89)		
Mode of conception				
Spontaneous	907 (88.83)	114 (11.17)	4.89	0.087
Promoting ovulation	8 (072.73)	3 (27.27)		
Artificial impregnation	16 (100.00)	0 (0.00)		
Number of pregnancies				
Once	460 (91.17)	44 (8.73)	7.96	0.047
Twice	302 (85.55)	51 (14.15)		
3 to 5 times	162 (89.01)	20 (10.99)		
More than 5 times	7 (77.78)	2 (22.22)		
Number of deliveries				
Once	622 (89.37)	74 (10.63)	1.05	0.789
Twice	277 (87.93)	38 (12.06)		
3 to 5 times	30 (85.71)	5 (14.29)		
More than 5 times	2 (100.00)	0 (0.00)		
Adverse pregnancy history				
None	793 (89.71)	91 (10.29)	7.1	0.213
Premature labour	10 (90.91)	1 (9.09)		
Abortion	73 (84.88)	13 (15.12)		
Exfetation	17 (89.47)	2 (10.53)		
Overdue	5 (71.43)	2 (28.57)		
Others	33 (80.49)	8 (19.51)		
High-risk maternity				
Yes	143 (86.67)	22 (13.33)	0.93	0.335
No	788 (89.24)	95 (10.76)		
How many kids is that?				
A child	604 (89.61)	70 (10.39)	2.64	0.451
Second child	302 (87.79)	42 (12.21)		
Third child	22 (81.48)	5 (18.52)		
Fourth child and above	3 (100)	0 (0.00)		
Postpartum stage				
6 weeks	483 (91.13)	47 (8.87)	33.34	<0.001
6-8 weeks	156 (96.30)	6 (3.70)		
8 weeks-6months	190 (84.82)	34 (15.18)		
7-12 months	102 (77.27)	30 (22.73)		
Fatal sex				
Boy	446 (89.02)	55 (10.98)	13.19	0.01
Girl	472 (89.39)	56 (10.61)		
Same gender twins	4 (50.00)	4 (50.00)		
Different gender twins	3 (75.00)	1 (25.00)		
Other	6 (85.71)	1 (14.29)		
Psycho and social characteristics				
Your fetal sex expectation				
Boy	63 (82.89)	13 (17.11)	12.81	0.002
Girl	103 (81.10)	24 (18.90)		
No difference	765 (90.53)	80 (9.47)		
Consistency in fetal sex expectation				
Yes	816 (90.56)	85 (9.43)	19.39	<0.001
No	115 (78.23)	32 (21.77)		
Key family members' fetal sex expectation				
Boy Better	55 (69.62)	24 (30.38)	51.54	<0.001
Girl Better	78 (78.23)	23 (22.77)		
No difference	798 (91.94)	70 (8.06)		
Postpartum childcare centers?				
Yes	134 (89.93)	15 (10.07)	0.21	0.646
No	797 (88.65)	102 (11.35)		
Know postpartum depression?				
Yes	511 (91.41)	48 (8.59)	8.02	0.005
No	420 (85.89)	69 (14.11)		
Know where seeking-help for postpartum depression				
Yes	386 (94.46)	27 (6.54)	14.71	<0.001
No	545 (85.83)	90 (14.17)		

*p* < 0.05 there was a statistically significant difference.

### 3.1 Associated characteristics of women with PPD symptoms

**Table 1** summarizes the differences between variables with and without depression symptoms. The chi-square test results presents the demographic, health-related, and obstetric characteristics of women with postpartum depression (PPD) symptoms, categorized by their PHQ-9 scores (PHQ-9 < 10 and PHQ-9 ≥ 10). The chi-square test results of PHQ-9 scores on various variables are presented in **Table 1**. Women with primary education or below had a significantly higher proportion of PPD symptoms (26.09%) compared to those with higher education levels (χ2 = 20.27,p < 0.001). Women from families with only one child showed a lower proportion of PPD symptoms (9.07%) compared to those with more than one child (12.82%), though the difference was marginally significant (χ2 = 3.66,p > 0.056). Women living with their partners only had a lower proportion of PPD symptoms (9.43%) compared to those living with parents or in-laws (13.06%) or those in long-distance relationships (23.08%) χ^2^ = 10.60,p < 0.05). Women who never consumed alcohol had a lower proportion of PPD symptoms (10.40%) compared to those who used to drink but quit (21.87%)(χ^2^ = 8.21,p < 0.05). Women who exercised regularly had a lower proportion of PPD symptoms (7.07%) compared to those who never exercised (14.73%) (χ^2^ = 8.28,p < 0.05). Women with planned pregnancies had a lower proportion of PPD symptoms (9.08%) compared to those with unplanned pregnancies (15.89%) (χ ^2^ = 10.41,p < 0.01). Women in the 6–8 weeks postpartum stage had the lowest proportion of PPD symptoms (3.70%), while those in the 7–12 months stage had the highest (22.73%) (χ^2^ = 33.34,p < 0.001). Women who had no specific expectation for the fetal sex had a lower proportion of PPD symptoms (9.47%) compared to those who expected a boy (17.11%) or a girl (18.90%) (χ^2^ = 1.81,p < 0.01).Women who knew about postpartum depression had a lower proportion of PPD symptoms (8.59%) compared to those who did not (14.11%) (χ^2^ = 8.02,p < 0.01).*3.2. Sociodemographic characteristics on the WEM-WBS and MPS*.

**Table 2** examines the association between various demographic and health-related variables and the WEM-WBS (Well-being) and MPS (Marital Satisfaction) scores. The mean score on the WEM-WBS was 55.54 ± 12.041 (total possible score = 70) and the mean score on the MHS was 77.955 ± 24.586 (total possible score = 100).Women married for less than 1 year had higher WEM-WBS scores (56.77 ± 11.82) compared to those married for 5 years or more (53.07 ± 12.76) (*F* = 4.27, *p* < 0.01). Women with higher incomes (≥ 10000 RMB) had higher WEM-WBS scores (56.93 ± 11.86) compared to those with lower incomes (≤ 3000 RMB) (53.10 ± 12.93) (*F* = 4.93, *p* < 0.01). Urban women had higher WEM-WBS scores (56.54 ± 11.63) compared to rural women (53.08 ± 12.69) (*t* = 4.1, *p* < 0.001).Women living with their partners only had higher WEM-WBS scores (56.75 ± 11.8) compared to those living with parents or in-laws (53.53 ± 12.16) (*F* = 8.2, *p* < 0.001). Women who exercised regularly had higher WEM-WBS scores (59.65 ± 9.84) compared to those who never exercised (52.40 ± 12.73)(*F* = 25.25, *p* < 0.001). Women with planned pregnancies had higher WEM-WBS scores (56.65 ± 11.74) compared to those with unplanned pregnancies (53.03 ± 12.36) (*t* = 4.43, *p* < 0.001). Women in the 6–8 weeks postpartum stage had the highest WEM-WBS scores (59.61 ± 10.90), while those in the 7–12 months stage had the lowest (54.27 ± 14.10) (*F* = 7.56, *p* < 0.001).

**Table 2:** Sociodemographic characteristics on the WEM-WBS and MPS.

Variables	Number	WEM-WBS score			MPS score		
	N (%)	Mean±SD	F/t	*p*	Mean±SD	F/t	*p*
	1048	55.54±12.04			77.96±24.59		
Demographic							
Years of marriage							
<1 year	363 (34.64)	56.77±11.82	4.27	0.005	80.939±23.67	3.39	0.018
1~2 years	315 (30.06)	56.04±11.93			75.08±25.84		
3~4 years	200 (19.08)	54.62±11.68			78.35±21.91		
≥5 years	170 (16.22)	53.07±12.76			76.447±26.50		
Educational level							
Primary and below	46 (4.39)	50.8±15.34	4.57	0.001	66.44±30.27	4.96	0.001
Lower or upper secondary school	441 (42.08)	54.56±11.56			76.02±24.61		
Technical college	303 (28.91)	56.77±11.37			80.60±23.22		
Bachelor	241 (23.00)	56.30±12.83			79.76±24.76		
Postgraduate or above	17 (1.62)	61.29±8.42			86.59±14.44		
Only child family							
Yes	463 (44.18)	55.57±12.55	0.07	0.947	78.25±25.08	0.35	0.73
No	585 (55.82)	55.52±11.63			77.72±24.20		
Monthly household income							
≤3000 RMB	261 (24.90)	53.10±12.93	4.93	0.002	74.33±26.02	3.1	0.027
3001-4999 RMB	333 (31.37)	56.16±11.10			77.96±24.28		
5000-9999 RMB	366 (34.92)	56.39±12.06			80.50±23.53		
≥ 10000 RMB	88 (8.40)	56.93±11.86			78.10±24.75		
Location							
Rural	303 (28.91)	53.08±12.69	4.1	<0.001	72.00±26.55	4.79	<0.001
Urban	745 (71.09)	56.54±11.63			80.38±23.33		
Cohabitation							
Partner Only	689 (65.74)	56.75±11.8	8.2	<0.001	80.829±23.186	12.2	<0.001
Parents (or in-law)	268 (25.57)	53.53±12.16			74.42±25.03		
Long-distance husband and wife	52 (4.96)	50.39±11.96			64.12±29.55		
Others	39 (3.72)	54.80±11.92			69.95±27.96		
Working consistently during pregnancy							
Yes	223 (21.28)	57.27±12.64	2.43	0.015	78.18±25.48	0.15	0.878
None	825 (78.72)	55.07±11.84			77.90±24.35		
Health-related							
Smoking							
Never	967 (92.27)	55.73±11.91	1.63	0.197	78.58±24.02		
Used to smoke, quitting	50 (4.77)	53.42±12.63			72.20±29.83	3.01	0.058
Occasional smokers (more than once a month)	31 (2.96)	52.94±14.81			67.74±29.71		
Alcohol consumption							
Never	971 (92.65)	55.89±11.82	7.27	0.001	78.80±24.02	6.28	0.006
Used to drink, Cut out now	64 (6.11)	50.02±13.71			65.20±29.73		
Occasional drinkers (multiple times per month)	13 (1.24)	56.39±14.13			77.31±23.22		
Exercise							
Never	353 (33.68)	52.40±12.73	25.25	<0.001	70.90±26.00	23.77	<0.001
Occasionally (more than once a month)	497 (47.42)	56.13±11.77			80.41±23.37		
Regular (more than once a week)	198 (18.89)	59.65±9.84			84.37±22.00		
Obstetric, current pregnancy, and infant-related characteristics, and their association							
Planned pregnancy							
Yes	727 (69.37)	56.65±11.74	4.43	<0.001	80.62±23.00	5.03	<0.001
No	321 (30.63)	53.03±12.36			71.93±26.93		
Mode of conception							
Spontaneous	1021 (97.42)	55.626±12.038	2.87	0.057	78.18±24.46	2.96	0.052
Promoting ovulation	11 (1.05)	46.91±12.45			60.27±38.01		
Artificial impregnation	16 (1.53)	56.00±10.30			75.81±17.88		
Number of pregnancies							
Once	504 (48.09)	56.24±12.34	2.21	0.085	81.28±23.34	7.99	<0.001
Twice	353 (33.68)	55.59±12.15			76.69±24.80		
3 to 5 times	182 (17.37)	53.67±10.81			71.32±26.11		
More than 5 times	9 (0.86)	52.67±11.50			75.44±24.55		
Number of deliveries							
Once	696 (66.41)	55.10±12.33	1.34	0.26	78.18±24.41	0.58	0.632
Twice	315 (30.06)	56.66±11.26			77.90±24.72		
3 to 5 times	35 (3.34)	54.31±12.49			75.09±26.83		
More than 5 times	2 (0.19)	56.00±19.80			59±39.60		
Adverse pregnancy history							
None	884 (84.35)	56.27±11.97	5.16	<0.001	79.30±24.28	5.04	<0.001
Premature labour	11 (1.05)	58.09±10.30			87.82±14.70		
Abortion	86 (8.21)	51.62±11.61			70.21±25.08		
Exfetation	19 (1.81)	51.95±12.45			72.05±24.84		
Overdue	7 (0.67)	47.00±9.42			77.71±24.81		
Others	41 (3.91)	50.49±12.04			72.05±24.84		
High-risk maternity							
Yes	165 (15.74)	54.66±13.20	1.02	0.307	74.82±26.70	1.79	0.074
No	883 (84.26)	55.70±11.81			78.54±24.14		
How many kids is that?							
A child	674 (64.31)	55.15±12.33	1.83	0.14	78.39±24.19	0.67	0.574
Second child	344 (32.82)	56.50±11.39			77.52±25.04		
Third child	27 (2.58)	52.33±12.62			72.04±29.39		
Fourth child and above	3 (0.29)	61.33±7.57			82.67±10.26		
Postpartum stage							
6 weeks	530 (50.57)	54.79±11.58	7.56	<0.001	77.38±23.92	6.54	<0.001
6-8 weeks	162 (15.46)	59.61±10.90			84.90±21.57		
8 weeks-6months	224 (21.37)	55.12±12.07			76.25±25.41		
7-12 months	132 (12.60)	54.27±14.10			74.62±27.79		
Fatal sex							
Boy	501 (47.81)	55.14±12.37	4.638	0.001	76.84±25.80	5.16	<0.001
Girl	528 (50.38)	56.29±11.55			79.73±23.04		
Same gender twins	8 (0.76)	41.88±11.78			46.00±21.47		
Different gender twins	4 (0.38)	52.00±16.51			75.00±30.41		
Other	7 (0.67)	45.29±9.32			62.00±18.91		
Psycho and social characteristics							
Your fetal sex expectation							
Boy	76	53.00±14.17	9.82	<0.001	68.49±30.47	7.54	0.001
Girl	127	51.80±13.45			72.87±28.01		
No difference	845	56.33±11.48			79.57±23.14		
Consistency in fetal sex expectation							
Yes	901 (85.97)	56.38±11.79	5.64	<0.001	79.40±23.68	4.21	<0.001
No	147 (14.03)	50.42±12.32			69.102±28.04		
Key family members' fetal sex expectation							
Boy Better	79 (7.54)	45.65±14.33	36.631	<0.001	58.03±30.68	21.89	<0.001
Girl Better	101 (9.64)	52.60±12.93			73.41±27.00		
No difference	868 (82.82)	56.78±11.22			80.30±22.74		
Postpartum childcare centers?							
Yes	149 (14.22)	53.73±15.05	-1.99	0.047	73.86±28.70	-1.93	0.056
No	899 (85.78)	55.84±11.45			78.63±23.79		
Know postpartum depression?							
Yes	559 (53.33)	57.09±11.78	-4.49	<0.001	80.59±23.93	-3.73	<0.001
No	489 (46.67)	53.77±12.10			74.95±25.00		
Know where seeking-help for postpartum depression							
Yes	413 (39.41)	57.17±11.71	-3.56	<0.001	79.86±23.33	-2.06	0.04
No	635 (60.59)	54.48±12.14			76.72±25.31		

p < 0.05 there was a statistically significant difference.

The study examined the associations between various demographic, health-related, obstetric, and psychosocial characteristics with the Maternal Postnatal Scale (MPS) scores. The results are presented in **Table 2**, highlighting significant differences across multiple variables. Significant differences in MPS scores were observed based on the duration of marriage (*F* = 3.39, *p* < 0.05). Participants married for less than one year had higher MPS scores (80.94 ± 23.67) compared to those married for 1–2 years (75.08 ± 25.84), 3–4 years (78.35 ± 21.91), and 5 years or more (76.45 ± 26.50). Educational attainment significantly influenced MPS scores (*F* = 4.96, *p* < 0.001). Participants with postgraduate education or above had the highest MPS scores (86.59 ± 14.44), while those with primary education or below had the lowest scores (66.44 ± 30.27). Urban residents had significantly higher MPS scores (80.38 ± 23.33) compared to rural residents (72.00 ± 26.55) (*F* = 4.79, *p* < 0.001). Participants living only with their partners had the highest MPS scores (80.83 ± 23.19), while those in long-distance relationships had the lowest scores (64.12 ± 29.55) (*F* = 12.2, *p* < 0.001). In health-related characteristic,Non-smokers had higher MPS scores (78.58 ± 24.02) compared to occasional smokers (67.74 ± 29.71) (*F* = 3.01, *p* = 0.058). Non-drinkers had significantly higher MPS scores (78.80 ± 24.02) compared to occasional drinkers (65.20 ± 29.73) (*F* = 6.28, *p* < 0.001). Regular exercisers had the highest MPS scores (84.37 ± 22.00), while those who never exercised had the lowest scores (70.90 ± 26.00) (*F* = 23.77, *p* < 0.001).

### 3.3 Postpartum PHQ-9 score and differences on the WEM-WBS and MPS

**Figure 1:**
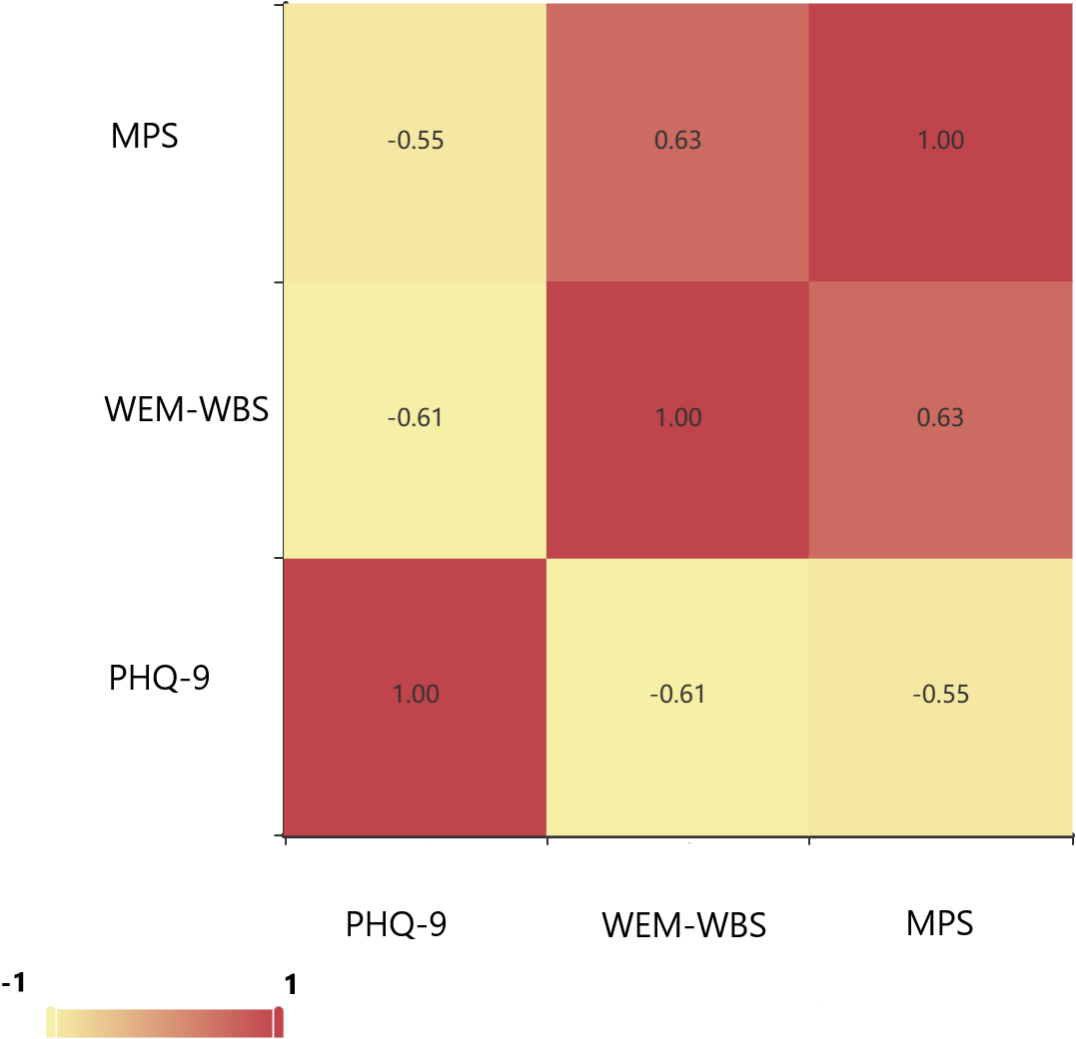
Associations between the PHQ-9 score, WEM-WBS, and MPS. PPD, Postpartum depression; ACOG, American College of Obstetricians and Gynecologists; PHQ-9, Patient Health Questionnaire-9; EPDS, Edinburgh Postpartum Depression Scale; WEM-WBS, Warwick-Edinburgh mental health scale; MPS, Marriage perception scale.

The results also revealed significant variations in the WEM-WBS and MHS scores across different levels of depressive symptoms (**Table 3**), as well as varying degrees of severity in depressive symptoms (**Table 4**). Women with PHQ-9 scores ≥ 10 had significantly lower WEM-WBS scores (39.81 ± 9.59) compared to those with PHQ-9 scores < 10 (57.52 ± 10.80) (*t* = -18.544, *p* < 0.001). Women with PHQ-9 scores ≥ 10 had significantly lower couple interaction scores (40.50 ± 15.04) compared to those with PHQ-9 scores < 10 (59.90 ± 13.24) ((*t* = -14.708, *p* < 0.001). Women with PHQ-9 scores ≥ 10 had significantly lower couple relationship scores (22.28 ± 7.84) compared to those with PHQ-9 scores < 10 (30.79 ± 6.60) ((*t* = -11.243, *p* < 0.001). Women with PHQ-9 scores ≥ 10 had significantly higher couple conflict scores (14.63 ± 6.39) compared to those with PHQ-9 scores < 10 (9.00 ± 5.27) ((*t* = 9.162, *p* < 0.001). Women with PHQ-9 scores ≥ 10 had significantly lower marriage happiness scores (48.15 ± 24.44) compared to those with PHQ-9 scores < 10 (81.70 ± 21.91) ((*t* = -14.153, *p* < 0.001)(**Table 3**). Specifically, women experiencing depressive symptoms exhibited poorer positive psychological qualities, lower levels of marital happiness, and deteriorated relationships characterized by reduced interaction and increased conflict (**Figure 1**). Furthermore,Women with severe PHQ-9 scores had the lowest WEM-WBS scores (37.38 ± 10.68), while those with no PHQ-9 symptoms had the highest (59.61 ± 10.14) (*F* =189.68, p < 0.001).Women with severe PHQ-9 scores had the lowest couple relationship scores (22.17 ± 8.79), while those with no PHQ-9 symptoms had the highest (31.65 ± 6.29) (*F* = 67.03,p < 0.001). Women with severe PHQ-9 scores had the highest couple conflict scores (15.23 ± 7.25), while those with no PHQ-9 symptoms had the lowest (8.21 ± 4.75) (*F* = 56.03, p < 0.001).Women with severe PHQ-9 scores had the lowest marriage perception scores (45.96 ± 28.28), while those with no PHQ-9 symptoms had the highest (85.34 ± 20.31) (*F* =110.88, p < 0.001) (**Table 4**).

**Table 3:** PHQ-9 score differences on the WEM-WBS and MPS.

Tools	PHQ-9 score	N (%)	Mean±SD	t	*p*
WEM-WBS	≥10	117 (7.91)	39.81±9.59	-18.544	<0.001
	<10	931 (92.09)	57.52±10.80		
	All	1048 (100)	55.54±12.04		
Couple's interaction	≥10	117 (7.91)	40.50±15.04	-14.708	<0.001
	<10	931 (92.09)	59.90±13.24		
	All	1048 (100)	57.74±14.77		
Couple‘s relationship	≥10	117 (7.91)	22.28±7.84	-11.243	<0.001
	<10	931 (92.09)	30.79±6.60		
	All	1048 (100)	29.84±7.26		
Couple's conflict	≥10	117 (7.91)	14.63±6.39	9.162	<0.001
	<10	931 (92.09)	9.00±5.27		
	All	1048 (100)	9.62±5.69		
Marriage happiness	≥10	117 (7.91)	48.15±24.44	-14.153	<0.001
	<10	931 (92.09)	81.70±21.91		
	All	1048 (100)	77.96±24.59		

*p* < 0.05 there was a statistically significant difference.

**Table 4:** PHQ-9 degree differences on the WEM-WBS and MPS.

Tools	Degree of PHQ-9 score	N(%)	Mean±SD	Welch's F	*p*
WEM-WBS	None	761 (72.61)	59.61±10.14	189.681	<0.001
	Mild	70 (6.68)	41.44±8.48		
	Moderate	170 (16.22)	48.15±8.47		
	Severe	47 (4.48)	37.38±10.68		
	All	1048 (100)	55.54±12.04		
Couple's interaction	None	761 (72.61)	61.90±12.50	95.015	<0.001
	Mild	70 (6.68)	41.49±13.43		
	Moderate	170 (16.22)	50.95±12.80		
	Severe	47 (4.48)	39.02±17.20		
	All	1048 (100)	57.74±14.77		
Couple‘s relationship	None	761 (72.61)	31.65±6.29	67.026	<0.001
	Mild	70 (6.68)	22.36±7.20		
	Moderate	170 (16.22)	26.97±6.61		
	Severe	47 (4.48)	22.17±8.79		
	All	1048 (100)	29.84±7.26		
Couple's conflict	None	761 (72.61)	8.21±4.75	56.026	<0.001
	Mild	70 (6.68)	14.23±5.77		
	Moderate	170 (16.22)	12.50±6.03		
	Severe	47 (4.48)	15.23±7.25		
	All	1048 (100)	9.62±5.69		
Marriage perception	None	761 (72.61)	85.34±20.31	110.882	<0.001
	Mild	70 (6.68)	49.61±21.58		
	Moderate	170 (16.22)	65.42±21.49		
	Severe	47 (4.48)	45.96±28.28		
	All	1048 (100)	77.96±24.59		

*p* < 0.05 there was a statistically significant difference.

### 3.4 Mediation analysis of the WEM-WBS score in the MPS score on the depression level

**Table 5** presents the results of a stepwise regression analysis examining the predictors of PHQ-9 scores After automatic recognition by the model, three items were included in the final model: the total WEM-WBS score; the marital relationship on the MPS; and the marital happiness score on the MPS. The R2 was 0.44, indicating that these variables explained 44% of variation in PHQ-9 scores(p< 0.001). WEM-WBS scores were negatively associated with PHQ-9 scores (β = -0.42, p < 0.001). Couple’s relationship scores were positively associated with PHQ-9 scores (β = 0.44, p < 0.001).Marriage perception scores were negatively associated with PHQ-9 scores (β = -0.70, p < 0.001).D-W was 2.0.4 generally considered as no significant autocorrelation (**Table 5**).

**Table 5:** Stepwise regression analysis (n = 1048).

Independent variables	B	SE	*β*	*t*	*p*
value	14.6	0.69	-	21.33	<0.001
WEM-WBS	-0.17	0.01	-0.42	-13.99	<0.001
Couple’s relationship(MPS)	0.29	0.05	0.44	5.99	<0.001
Marriage perception(MPS)	-0.14	0.02	-0.7	-9.01	<0.001
*R^2^*	0.44				
Adjusted *R^2^*	0.44				
F(*p*)	271.42( *p*<0.001)				
D-W value	2.04				

*p* < 0.05 there was a statistically significant difference.

**Table 6** presents the mediation analysis results for the relationship between PHQ-9 scores and couple’s conflict (MPS) and interaction (MPS). WEM-WBS scores were negatively associated with PHQ-9 scores (*β* = -0.61, *p* < 0.001) and positively associated with couple’s interaction (*β* = 0.61, *p* < 0.001). Couple’s conflict was negatively associated with PHQ-9 scores (*β* = -0.19, *p* < 0.001). Couple’s interaction was positively associated with PHQ-9 scores (*β* = 0.18, *p* < 0.001). The models explained 38% to 43% of the variance in the outcomes (Adjusted *R²* = 0.37 to 0.43, *p* < 0.001).

**Table 6:** PHQ and MPS mediation analysis.

Independent variables	PHQ-9 Score					Couple's conflict(MPS)					Couple's interaction(MPS)					PHQ-9 Score				
	B	SE	*t*	*p*	*β*	B	SE	*t*	*p*	*β*	B	SE	*t*	*p*	*β*	B	SE	*t*	*p*	*β*
Value	16.86	0.56	30.32	<0.001	–	16.03	1.7	9.41	<0.001	–	20.47	0.76	27.06	<0.001	–	14.73	0.74	20	<0.001	–
WEM-WBS	-0.25	0.01	-25.06	<0.001	-0.61	0.75	0.03	25.04	<0.001	0.61	-0.2	0.01	-14.67	<0.001	-0.41	-0.17	0.01	-14.02	<0.001	-0.42
Couple's conflict(MPS)																-0.06	0.01	-6.22	<0.001	-0.19
Couple's interaction(MPS)																0.15	0.02	6.87	<0.001	0.18
*R^2^*	0.38					0.38					0.17					0.43				
Adjusted *R^2^*	0.38					0.37					0.17					0.43				
*F(p)*	628.14( p<0.001)					626.95( p<0.001)					215.23( p<0.001)					267.26( p<0.001)				


*p* < 0.05 there was a statistically significant difference.

The mediating effects ratios of the MPS couple interaction and MPS couple conflict were 12.12% and 18.73%, respectively.

## 4 Discussion

In the current study the positive rate of depressive symptoms (PHQ-9 ≥ 10) in parturients was 11.16%. Wang Zy et al.’s 2021 systematic review of global studies revealed that meta-regression analysis indicated a pooled prevalence of PDD at 17.22% (45). This review encompassed 565 articles(prevalence of postpartum depression in the range of 3.5%–63.3%) published before July 2021, thoroughly examining regional and national prevalence differences, variations in assessment tools, and significant risk factors. The latest research findings exhibit significant variability depending on the geographical location and timing of the investigations.A study in Iran showed that the prevalence of PPD was 22.4% and 3.2%. at 3 days and at 6 month after delivery, respectively (7). Amer SA et al.’s multinational study, conducted across six countries - Egypt, Yemen, Iraq, India, Ghana, and Syria - revealed an overall postpartum depression (PPD) prevalence of 13.6%. The lowest prevalence was observed in Syria at 2.3%, while the highest was reported in Ghana at 26% (8). Xiao et al. and Xiaojuan et al. reported positive rates for depressive symptoms of 8.90% (46) and 7.69% in China, respectively (47), which were lower than our findings. A study conducted in Japan reported that the prevalence rates of postpartum depression (PPD) among mothers were 6.5% at 3 days, 7.5% at 3 months, 7.5% at 6 months, and 8.4% at 1 year postpartum, all of which were lower than the corresponding rates observed in our study (48). The distinction lies in the use of EPDS as opposed to our the PHQ-9. A retrospective study published after July 2021, involving 4,619 participants in the United States, revealed that 10.7% of postpartum women scored above 10 on the PHQ-9, indicating symptoms of postpartum depression. This investigation focused on women who had given birth in obstetric hospitals (49). Most studies primarily focus on the prevalence of symptoms measured by standardized scales rather than the prevalence of the clinical diagnosis.Furthermore, the depression diagnosis rate (approximately 9.0%) using the Structured Clinical Interview for DSM Disorders (SCID) was closely aligned with the EPDS at a score ≥10 (50, 51). As a result, measurements using self-report questionnaires will be higher than actual DSM diagnoses.

There are numerous factors that influence symptoms of PPD, including sociodemographic, physical and biological, cultural, and obstetric and pediatric factors (52). In the current study we used a comprehensive survey based on demographic,health-relatedObstetric, current pregnancy, and infant-related, Psycho and social characteristics. The study results revealed that significant variations were noted in the prevalence of depressive symptoms among women with respect to education level, co-habitation, alcohol consumption, exercise routine, number of pregnancies, fetal gender preference, and gender expectations.

The study findings indicated that individuals with lower educational attainment exhibit higher rates of PPD positivity. Few studies have determined the risk of education on PPD symptoms (53–55). However, other studies have shown that PPD symptoms are not related to education level (19, 56, 57).

A study indicated that unplanned pregnancy and first-time motherhood are risk factors for PPD (19). Our findings indicated that unplanned pregnancies exhibit a higher prevalence and associated score. However, this observation does not apply to first-time mothers.

Co-habitation status was shown to be a significant risk factor for PPD symptoms. The findings indicated that couples residing independently exhibited the lowest depressive symptom prevalence (9.87%). Several studies have specifically highlighted living with in-laws as a notable risk factor for PPD symptoms (31, 58). Co-habitants can influence PPD symptoms, which has often been the focus of research. Our study focused solely on the identities of the co-habitants and did not inquire into the depth of the relationships between the study participants and co-habitants. In this study we did not distinguish between parents and parents-in-law.

There was no significant association between newborn gender and the score or prevalence of depressive symptoms in parturients. Rihua et al. reported a higher prevalence of depressive symptoms among women in China who gave birth to girls (12.2%) compared to women who gave birth to boys (24.6%) (27). However, no significant difference was detected in postpartum rates between boys and girls. Interestingly, the presence of twins versus having a singleton did have a notable distinction, suggesting that the stress associated with childrearing may be influenced by the number of children. Studies have demonstrated that parturients who give birth to male infants face an increased susceptibility to depression, with the likelihood of experiencing PPD symptoms increasing by 105% in ACCESS and 424% in PRISM (59). Furthermore, mothers of boys exhibit a higher prevalence of depressive symptoms, which is possibly attributed to pro-inflammatory cytokines (60), reproductive hormone fluctuations (61), and maternal immune activation (62). Our research focuses more on social and cultural factors. This viewpoint will be discussed in detail below. We focused on fetal gender, maternal and familial expectations of fetal gender, as well as various scenarios in which there is consistency between fetal gender and expectations. The findings revealed that the level and prevalence of depressive symptoms in parturients were influenced by disparities between expected and actual fetal gender, as well as differences between expected fetal gender and the expectations held by immediate family members. Given the cultural significance attached to fetal gender in China, coupled with declining birth rates resulting from family planning policies favoring single-child households, it is plausible that gender expectations are linked to depressive symptoms. The current study demonstrated a significant disparity in depressive symptom scores between individuals who had specific gender preferences for their unborn child versus individuals without such preferences (independent of the desired gender). Interestingly, the newborn actual gender did not appear to be directly associated with maternal depressive symptoms. However, incongruence between anticipated and actual newborn gender significantly impacted maternal mental health outcomes. Furthermore, expectant mothers desiring a male offspring exhibited higher levels of depressive symptoms compared to mothers without specific gender preferences. Conversely, individuals who did not have a preference regarding newborn gender displayed lower levels of depressive symptoms. Therefore, we failed to identify any significant correlations among the aforementioned factors. Fetal gender satisfaction is a risk factor for PPD (63). A study conducted in Shenzhen, China also demonstrated that fetal gender and gender expectations have an impact on PPD symptoms (19).

We concur that the impact of factors, such as living arrangements, relationships with in-laws, fetal gender, and gender expectations on PPD symptoms, among women is closely tied to Chinese cultural norms, including the preference for male offspring, the traditional concept of filial piety in which in-laws are often regarded as having greater childrearing experience and thus deserve deference, and the potential for intergenerational conflicts between couples and their parents or in-laws (19). Cigarette smoking (64) and alcohol consumption (8) are recognized as risk factors for the development of PPD. Our findings indicated a significant association between PPD and regular alcohol consumption; no correlation was detected with cigarette smoking. This discrepancy may be attributed to the participants’ awareness of the detrimental effects of cigarette smoking on fetal health, leading to intentional reduction or cessation of smoking and rendering the impact statistically insignificant.

Prenatal and postnatal exercise should serve as a protective factor against PPD. Our findings indicated that exercise habits, including sporadic physical activity, are positively associated with a reduction in the symptoms and prevalence of PPD. These results align with most existing research in this field. Exercise has beneficial effects on the improvement of maternal depression and postpartum interventions may be more effective than antepartum interventions (65). Randomized controlled trials have shown that compared to no exercise, exercise-only interventions reduce the severity of PPD symptoms (66). Higher levels of physical activity during pregnancy are consistently associated with improved postpartum mental health outcomes, including reduced depressive symptoms, lower anxiety, and enhanced overall well-being (67).

We used to questions (“Know postpartum depression?” and “Know where to seek help for PPD?”) to determine the correlation between literacy and PPD symptoms. The research findings indicated that individuals with a higher awareness of PPD, as well as an understanding of the need for help and access to resources, exhibit lower depressive symptom scores and a reduced prevalence of depressive symptoms. Several studies have demonstrated that the level of PPD literacy among women in China is relatively low and it is imperative to implement measures to enhance the literacy of parturients (68, 69). Mental health literacy is positively associated with attitudes toward professional psychological help-seeking (70).

The current study investigated the impact of positive mental health and marital well-being on PPD in women by analyzing the correlations and significant differences in positivity and severity of depression. The findings indicated that the depressive symptom score was positively associated with mental well-being. Mental well-being is closely related to mental health symptoms. The Short Warwick-Edinburgh Mental Well-being Scale (SWEMWBS) could be used as an alternative to the PHQ-9 in monitoring and evaluating CMD treatment (71). There is evidence that poor mental well-being is associated with negative feelings, such as psychological stress, low self-esteem, loneliness, and depression (72). Positive psychology (PP) interventions are effective in reducing the level of depression and increasing mental well-being (73). A study conducted during the COVID-19 pandemic showed that deterioration in mental well-being makes the population vulnerable to several psychiatric conditions (74). There has been a paucity of in-depth research examining the relationship between mental well-being and depression, especially among women with PPD, and includes an exploration of potential causal relationships between mental health and PPD symptoms, as well as the role of mediating factors.

Ultimately, the MPS, which was developed for Chinese populations, was used to investigate the impact of marital happiness on PPD symptoms. The findings indicated a positive correlation between PPD symptoms and the overall score of marital happiness, as well as the subscales of marital interaction and marital relationship. Conversely, there was a negative correlation with marital conflict. Significant differences in the prevalence and severity of depressive symptoms were observed across various dimensions of marital happiness and its subscales.

A poor marital relationship, poor living conditions, and a lack of social support (63). Social support from the husband and family is significantly associated with PPD and this relationship may be modulated by fetus gender and the family’s expectations regarding fetal gender (63). A strained marital relationship increases the risk of PPD (75). The mother’s level of family cohesion, marital satisfaction, and parent-child relationship is closely associated with that of her partner. At 6 weeks postpartum, mothers who exhibit higher levels of family cohesion and lower levels of depressive symptoms demonstrate less impaired mother-child relationships (76).Marital satisfaction plays a crucial role in the development of perinatal depression both directly and indirectly through the mechanisms of family support and emotional connection. The greater the family support received by a gravida and parturient, the lower the EPDS score (77). The most significant factors contributing to elevated postpartum EPDS scores were higher prenatal EPDS scores, followed by inadequate communication between partners during pregnancy (the wife feeling unappreciated by her husband), and a lack of support from the husband postpartum (78). Despite the differences in the tools utilized in our study compared to other studies, marital quality, encompassing relationship dynamics, family support, and effective communication between partners, serves as protective factors against PPD. Conversely, poor marital quality, inadequate family support, and suboptimal interactions are identified as risk factors for PPD.

We performed an analysis to examine the impact of co-habitation arrangements on marital quality and the role as a covariate in PPD symptoms. The findings indicated that co-habitation arrangements significantly influenced marital happiness, marital conflict, marital interaction, and family relationships but did not have a combined effect on PPD symptoms. It is noteworthy that the study stands as the sole investigation to have examined the impact of mental well-being on PPD symptoms via the mediation of marital quality. However, delving deeper into the mechanisms underlying this influence or exploring additional relationships presents considerable challenges.

## 5 Limitations

This study was conducted in northeast China and provides highly valuable insight into the risk factors associated with PPD. Areas, such as literacy, mental well-being, and marital quality, are currently under-researched and further exploration of these related factors is constrained. However, This study did not choose to calculate the sample size, the convenience sampling method used in this study also limited the extrapolation of the results. In this study, we utilized the PHQ-9 questionnaire, although the majority of international studies predominantly employ the Edinburgh Postnatal Depression Scale (EPDS). Despite this difference, the PHQ-9 and EPDS have been shown to exhibit high consistency in their assessments. Self-report measurements can indicate the prevalence of depression symptoms but do not necessarily reflect the prevalence of a major depression disorder. It is widely acknowledged that PPD may be associated with various biological, genetic, hormonal, and other mechanistic changes. However, as a cross-sectional study, we were unable to comprehensively analyze the underlying mechanisms of these factors.

## 6 Conclusion

The findings of this study indicate a higher prevalence of depressive symptoms among postpartum women, influenced by multiple factors, particularly those associated with positive mental health and subjective marital satisfaction. However, the primary objective of postpartum depression screening is not merely to determine the prevalence of symptoms but to effectively reduce both the incidence of postpartum depression and its impact on affected women. From a tertiary prevention perspective, we recommend enhancing the dissemination of perinatal mental health knowledge to improve mental health literacy and awareness among perinatal women, with targeted health education addressing identified risk factors. Secondly, based on screening outcomes, it is crucial to provide tailored interventions for varying levels of symptom severity, including psychological counseling and psychotherapy. Women with more severe symptoms should be referred to psychiatry for comprehensive evaluation and treatment, which may involve pharmacotherapy, physical therapy, or cognitive-behavioral therapy (CBT). Thirdly, our study revealed that many symptomatic women are reluctant to accept recommended follow-up interventions. Investigating the barriers to help-seeking behavior is essential to identify and address these obstacles. Fourthly, research indicates that positive psychology and marital satisfaction are associated with reduced depressive symptoms. Therefore, promoting positive mental health and family well-being could serve as an effective preventive measure and warrants further investigation. Finally, from a broader healthcare system perspective, there is currently a lack of mental health services in obstetrics and gynecology departments in China. Efforts are underway to establish more psychological clinics within these departments and enhance mental health services. With ongoing policy support, we anticipate significant improvements in the tertiary prevention of perinatal mental health.

## Data Availability

The original contributions presented in the study are included in the article/supplementary material. Further inquiries can be directed to the corresponding author/s.
